# Ocrelizumab-Induced Hemophagocytic Lymphohistiocytosis: A Case Report

**DOI:** 10.7759/cureus.83102

**Published:** 2025-04-27

**Authors:** Tajudeen Musbau, Pyae Phyo Thinn, Hanusha Jeyanithi

**Affiliations:** 1 Hematology, Victoria Hospital, Kirkcaldy, Kirkcaldy, GBR; 2 Medicine, Victoria Hospital, Kirkcaldy, Kirkcaldy, GBR

**Keywords:** anakinra, anti-cd20, hlh, h-score, methylprednisolone, monoclonal antibody, multiple sclerosis, ocrelizumab, uclh

## Abstract

Hemophagocytic lymphohistiocytosis (HLH) is an uncommon disorder marked by severe immune system dysfunction and excessive inflammation. Its clinical features often mimic those of severe sepsis, including persistent high fevers, multiorgan failure, cytopenia, and coagulation abnormalities. HLH can be triggered by infections, malignancies, rheumatological disorders, genetic factors, and medications, particularly those that cause immunosuppression. Ocrelizumab, an anti-CD20 monoclonal antibody used in the treatment of multiple sclerosis, has been associated with rare immune-mediated complications. In this case, the patient presented with generalized fatigue, fever, and neutropenia and was initially treated for neutropenic sepsis. Despite standard antibiotic therapy, there was no clinical improvement. A CT scan of the chest, abdomen, and pelvis revealed multiple lung nodules with surrounding ground-glass halos, suggestive of an invasive fungal infection, along with hepatosplenomegaly, raising concern for an underlying hematological malignancy. The patient was managed with intravenous antibiotics and antifungal therapy. However, due to persistent high fevers, markedly elevated ferritin levels (peaking at 20,695 ng/mL), and pancytopenia, an H-score for HLH was calculated, yielding a score of 302, indicating a greater than 99% probability of the condition. Based on these findings, intravenous anakinra (100 mg) was initiated. A bone marrow biopsy showed features suggestive of HLH but no evidence of hematological malignancy. The case was discussed at the HLH multidisciplinary team meeting with the HLH team at University College London Hospitals NHS Foundation Trust, and methylprednisolone (500 mg) was subsequently started. Consultations with the rheumatology and respiratory teams were also arranged. The patient's condition gradually improved, and she was discharged on Day 31 with a tapering course of prednisolone, continued oral antifungal therapy, and a follow-up PET scan scheduled. This case highlights the importance of recognizing HLH as a potential complication in patients receiving immunosuppressive therapies such as ocrelizumab. Prompt diagnosis and aggressive management are critical for improving outcomes and preventing further complications.

## Introduction

Hemophagocytic lymphohistiocytosis (HLH) is an uncommon condition characterized by profound immune system dysfunction and excessive inflammation. There are two primary forms of HLH: primary and secondary. Primary HLH is genetic, resulting from inherited mutations that impair the cytotoxic function of natural killer (NK) cells and cytotoxic T cells. Key genetic mutations associated with primary HLH include *PRF1*, *UNC13D*, *STX11*, and *RAB27A*. In contrast, secondary (acquired) HLH is triggered by various factors that cause overactivation of the immune system, leading to a cytokine storm. This storm is marked by elevated levels of IL-1, IL-6, IL-18, TNF-α, and IFN-γ, which promote macrophage activation and subsequent hemophagocytosis [1].

Secondary HLH can be triggered by infections, malignancies, rheumatological disorders, and medications, particularly those that induce immunosuppression. Its clinical presentation often mimics severe sepsis, including persistent high fevers, multiorgan failure, cytopenia, and coagulation abnormalities. The estimated one-year mortality rate is approximately 50% [2], underscoring the importance of timely identification and intervention to improve patient outcomes.

Ferritin is a key diagnostic marker for HLH and should be measured early in patients presenting with persistent fever and systemic illness [3]. A ferritin level exceeding 10,000 ng/mL has been shown to be a highly sensitive and specific diagnostic indicator [4]. The H-score is a valuable tool for estimating the probability of HLH when clinical suspicion arises and incorporates various factors, including the presence or absence of hemophagocytosis on bone marrow aspirate [5]. However, it is important to note that while hemophagocytosis is a hallmark feature of HLH, it is neither highly sensitive nor specific [3], as it may also be observed in other critically ill patients without HLH [6].

## Case presentation

A 43-year-old woman with a known history of multiple sclerosis presented to the admission unit with complaints of general malaise, breathlessness, mild cough, and fever lasting for one day. She reported a recent episode of ingrown pubic hair, for which she had received oral antibiotics and a needle aspiration performed by her GP. Since then, she had been feeling unwell. The patient was due for her six-monthly ocrelizumab injection during this period, but due to neutropenia, it was delayed, with her last dose administered six months prior to this presentation. Her regular medications included fluoxetine 20 mg OD, carbamazepine 100 mg PO OD, pregabalin 300 mg BD, folic acid 5 mg OD, omeprazole 20 mg OD, senna 15 mg OD, co-codamol 30/500 PRN QD, and Evorel Sequi topical (fortnightly). She had no known allergies except for Stemetil. On admission, her vital signs were stable, and the physical examination was unremarkable.

Initial laboratory and clinical investigations

**Figure 1:**
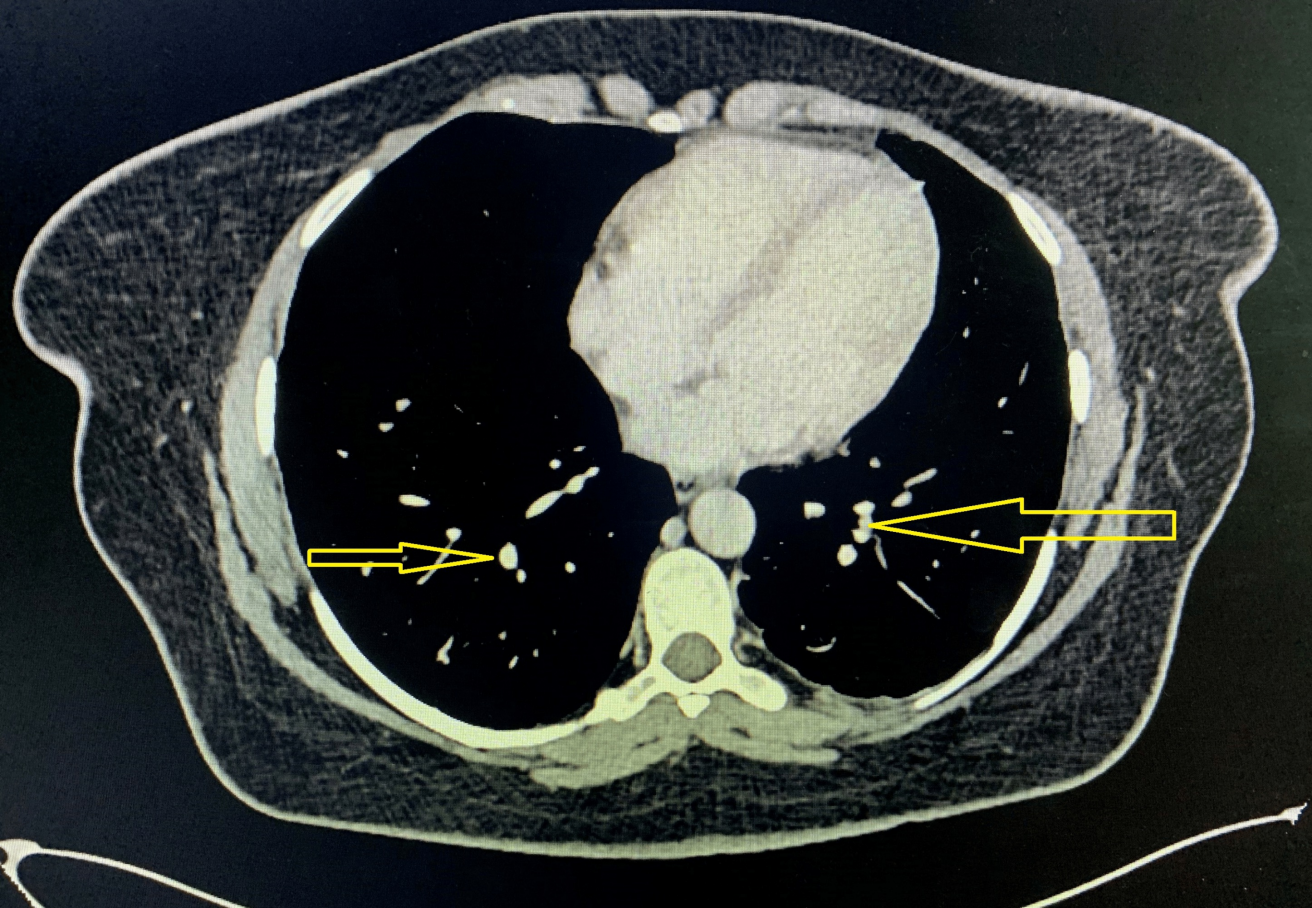
Chest CT scan showing multiple pulmonary nodules with surrounding ground-glass attenuation, suggestive of an invasive fungal infection

Initial laboratory results showed an absolute neutrophil count of 120/μL and a total WBC count of 1000/μL. CRP was elevated at 40.1 mg/L, with a hemoglobin level of 114 g/L and a platelet count of 166,000/μL. Other routine blood tests, including clotting profile, renal function, and liver function, were within normal limits. A septic screen was sent. Considering her neutropenia, she was started on intravenous tazobactam-piperacillin as per the neutropenic protocol on Day 1, and filgrastim 300 mcg was initiated on Day 3 of her admission. Carbamazepine was discontinued due to the neutropenia. Despite these interventions, she developed spiking fevers on Day 7 and complained of a severe, sharp frontal headache across the nasal bridge, which she attributed to a recent cold one month earlier. A CT of the chest, abdomen, and pelvis, performed on Day 10 of admission, revealed multiple pulmonary nodules with surrounding ground-glass attenuation, raising concerns for an invasive fungal infection, though there were no features of malignancy (Figure 1). Hepatosplenomegaly and subtle generalized sclerosis suggested a possible hematological malignancy. A CT sinus scan showed only features of chronic sinusitis. Antibiotics were escalated to meropenem, and caspofungin was added. Despite intensifying antibiotic therapy, her fever persisted, and she required oxygen supplementation the next day.

On Day 12 of her admission, she was transferred to the hematology team, where further investigations were carried out, including a connective tissue disease (CTD) screen and a viral throat swab, which tested positive for enterovirus and rhinovirus. She also had multiple negative blood cultures; her sputum grew *Klebsiella *and *Candida*, and she underwent bronchoalveolar lavage, which showed negative fungal cultures and was negative for acid-fast bacilli. Cytology revealed respiratory epithelial cells, macrophages, and inflammatory cells, with no evidence of malignancy or identifiable organisms. Hepatitis A, B, C, and E serologies were negative, as were tests for HIV, Epstein-Barr virus, cytomegalovirus, adenovirus, and parvovirus.

Diagnosis of HLH

Her ferritin level was markedly elevated at 6,382 ng/mL, and she also exhibited high aspartate aminotransferase and triglycerides and low fibrinogen, along with pancytopenia. This led to a calculated H-score of 282, which correlated with a greater than 99% probability of HLH (Table 1). Based on these findings, she was started on intravenous anakinra (100 mg), and antifungal therapy was changed to AmBisome on Day 13. Vitamin K 10 mg was administered intravenously due to deranged prothrombin time/activated partial thromboplastin time. A bone marrow biopsy performed on Day 14 showed some evidence of HLH but no features of hematological malignancy. The case was discussed at the HLH multidisciplinary team meeting (MDTM) with the HLH team at University College London Hospitals NHS Foundation Trust, and methylprednisolone (500 mg) was initiated. Rheumatology and respiratory teams were also consulted.

**Table 1:** Daily H-score calculation AST, aspartate aminotransferase; HLH, hemophagocytic lymphohistiocytosis

Parameters	December 20	January 2	January 3	January 4	January 5	January 6	January 7	January 8	January 9	January 10	January 11	January 12	Reference range
Hemoglobin (g/L)	130	76	79	70	90	86	82	90	87	95	98	98	130-170 g/L
WBC (/μL)	1,000	800	1,900	2,400	2,200	2,400	6,600	9,700	7,700	5,300	4,800	4,200	4,000-10,000/μL
Platelets (/μL)	153,000	15,000	16,000	8,000	10,000	22,000	27,000	50,000	90,000	80,000	88,000	87,000	150,000-410,000/μL
AST (U/L)	-	249	-	-	136	-	51	-	32	-	-	23	10-45 U/L
Fibrinogen (g/L)	4.1	2.3	1.8	1.3	1.1	-	0.9	-	1.2	1.8	-	2.3	2.0-6.0 g/L
Triglyceride (mmol/L)	-	3.66	4.13	-	5.97	-	4.88	6.15	4.79	4.81	5.21	5.9	0.85-2.0 mmol/L
Ferritin (μg/L)	-	6,382	20,696	-	5,547	3,831	1,840	-	1,559	-	-	1,480	13-150 μg/L
CRP (mg/L)	40	152	-	-	-	17	-	-	10.4	-	-	-	0.0-5.0 mg/L
H-score	-	282	302	253	238	238	228	228	228	204	204	185	0
HLH % probability	-	99	99	99	98	98	96	96	96	88	88	70	<1%

**Figure 2:**
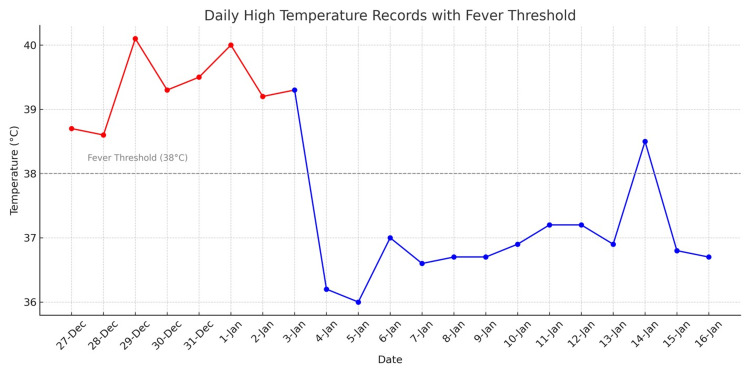
Daily highest temperature record

On Day 17, the patient developed oral cold sores and vaginal thrush, for which she was started on oral acyclovir and intravaginal Canesten. Rheumatology reviewed her case and found no signs of CTD, with the CTD screen returning negative. She improved clinically, as evidenced by the resolution of fever (Figure 2) and a progressive decline in her H-score. Meropenem was switched to co-amoxiclav for a further five days on Day 21. By Day 24, methylprednisolone was switched to oral prednisolone, and anakinra was discontinued. Over the next several days, her clinical condition continued to improve. She was clinically stable, allowing for prednisolone tapering starting on Day 27. Liposomal amphotericin B (AmBisome) was switched to oral voriconazole, with a planned total duration of six weeks and serum levels monitored twice weekly to maintain a therapeutic range of 2-5.5 mcg/mL. She was also supported with two units of red cell concentrate and three pools of platelets during this admission.

Outcome

She was discharged on January 20 after a prolonged hospitalization, with follow-up plans for continued monitoring of her neurological and infectious status. This case was discussed at the HLH University College London Hospitals NHS Foundation Trust MDTM, and it was concluded that HLH was triggered by a combination of ocrelizumab and severe infection. A PET scan on completion of steroids and antibiotics was suggested to fully exclude an underlying malignancy. Ocrelizumab was stopped, and neurology follow-up was arranged.

Follow-up

She had two further admissions post-discharge. The first admission was on January 22 with acute confusion due to severe hyponatremia (syndrome of inappropriate antidiuretic hormone secretion) and possible viral meningoencephalitis (raised protein in CSF and a nonspecific lesion in the occipital lobe), which was treated with intravenous acyclovir and ceftriaxone, along with hypertonic saline and oral slow sodium tablets for maintenance. Her second admission, on February 23, was for the treatment of *Escherichia coli *bacteremia, with the organism isolated from urine, sputum, and blood cultures. This infection was considered a complication resulting from immunosuppression.

## Discussion

HLH is a rare but life-threatening complication that can be triggered by various factors, including infections, malignancies, and medications. Ocrelizumab, an anti-CD20 monoclonal antibody used to treat multiple sclerosis, has been linked to rare immune-mediated complications.

The diagnosis of HLH can be confirmed when one of the following criteria is met: (1) a molecular diagnosis consistent with HLH, such as mutations in HLH-associated genes, or (2) five or more of the following eight clinical and laboratory criteria: fever >38.5°C, splenomegaly, cytopenia affecting ≥2 of 3 peripheral blood lineages, hypertriglyceridemia and/or hypofibrinogenemia, hemophagocytosis in bone marrow, spleen, liver, lymph nodes, or other tissues, low or absent NK cell activity, serum ferritin ≥500 μg/L, and elevated soluble CD25 (soluble IL-2 receptor α) ≥2,400 U/mL [7]. Our patient met six of these eight diagnostic criteria for HLH, including fever, organomegaly, cytopenia, hypertriglyceridemia, hypofibrinogenemia, elevated serum ferritin, and a background of immunosuppression.

This case emphasizes the importance of recognizing HLH in patients with a history of immunosuppressive therapy, particularly when there is evidence of systemic inflammation, pancytopenia, and elevated ferritin levels. Early recognition and appropriate management are crucial to improving patient outcomes. A search of the PubMed database revealed only one previously reported case with similar findings, making this patient the second documented case. The case, reported by Machlańska et al., involved a 32-year-old Caucasian female with multiple sclerosis treated with Ocrelizumab [8]. This underscores the rarity yet emerging recognition of HLH as a potential complication in patients undergoing Ocrelizumab therapy.

## Conclusions

Ocrelizumab-induced HLH is a rare but potentially life-threatening complication that demands high clinical suspicion for timely diagnosis and intervention. This case highlights the importance of monitoring for hyperinflammatory syndromes in patients undergoing B-cell-depleting therapies. Early recognition and appropriate management, including immunosuppressive therapy and discontinuation of the offending agent, are crucial to improving patient outcomes. As the use of ocrelizumab increases in autoimmune conditions, clinicians should remain vigilant for atypical presentations of HLH in this patient population.
